# Comparative gut microbiome in diarrheal and non-diarrheal children: an individually matched case-control study

**DOI:** 10.1128/msystems.00198-26

**Published:** 2026-09-30

**Authors:** Jialiang Xu, Yufeng Fan, Geruo Qu, Jiaqi Li, Yao Peng, Mengyu Wang, Jingyun Zhang, Yuqing Feng, Xiao Liu, Yongfei Hu, Biao Kan, Zhenpeng Li, Mei Zeng, Xin Lu

**Affiliations:** 1 School of Light Industry Science and Engineering, Beijing Technology and Business University 58276, Beijing, China; 2 Department of Clinical Laboratory, Shanxi Bethune Hospital, Shanxi Academy of Medical Sciences, Third Hospital of Shanxi Medical University, Tongji Shanxi Hospital, Taiyuan, China; 3 National Key Laboratory of Intelligent Tracking and Forecasting for Infectious Diseases, National Institute for Communicable Disease Control and Prevention, Chinese Center for Disease Control and Prevention and Chinese Academy of Preventive Medicine 96698, Beijing, China; 4 School of Public Health, Cheeloo College of Medicine, Shandong University 12589, Jinan, Shandong, China; 5 State Key Laboratory of Animal Nutrition and Feeding, College of Animal Science and Technology, China Agricultural University 34752, Beijing, China; 6 Department of Infectious Disease, Children’s Hospital of Fudan University, National Children’s Medical Center, Shanghai, China; BRIC National Agri-Food and Biomanufacturing Institute, Mohali, Punjab, India; BRIC National Agri-Food and Biomanufacturing Institute, Mohali, Punjab, India; Capital Institute of Pediatrics, Beijing, China

**Keywords:** individually matched case-control study, diarrheal children, non-diarrheal children, gut microbiota, metagenomics

## Abstract

Diarrhea, a leading cause of under-five mortality in developing countries, drives therapeutic challenges amid global antibiotic resistance. This study aimed to compare gut microbiota, antibiotic resistance genes (ARGs), mobile genetic elements (MGEs), and virulence factors (VFs) in diarrheal and non-diarrheal children, explore reasons for asymptomatic pathogen carriage, and develop a microbiome-based model to predict the potential etiology of diarrhea. This individually matched case-control study analyzed 42 paired fecal samples (selected from 716 diarrhea/non-diarrhea cases) to compare gut microbiome profiles, ARGs, MGEs, and VFs using metagenomic sequencing and pathogen-specific PCR. Diarrheal children showed reduced alpha diversity, increased Proteobacteria, and elevated VFs and MGEs. Surprisingly, ARGs were more abundant in non-diarrheal children, suggesting ARG colonization in healthy hosts. *Actinomyces* was enriched in non-diarrheal pathogen carriers and may be associated with asymptomatic bacterial pathogen carriage. A random forest (RF) model incorporating non-pathogenic bacteria achieved high accuracy in predicting diarrheal status and pathogen carriage. This study reveals distinct microbial ecologies between diarrheal and healthy children. The enrichment of specific bacterial taxa in asymptomatic carriers may be associated with pathogen tolerance. The unexpected ARG abundance in healthy children highlights a hidden antimicrobial resistance reservoir. These findings inform microbiome-based diagnostics and antibiotic stewardship in pediatric diarrhea.

**IMPORTANCE:**

This study is significant because it provides robust, individually matched case-control evidence linking childhood diarrhea to gut microbiome dysbiosis and the distribution of pathogenic and resistance-related genetic elements. By integrating metagenomic sequencing with pathogen-specific polymerase chain reaction (PCR), it offers a comprehensive comparison of microbial composition, virulence factors (VFs), mobile genetic elements (MHEs), and antibiotic resistance genes (ARGs) between diarrheal and non-diarrheal children. The findings demonstrate reduced microbial diversity and enrichment of Proteobacteria, virulence factors, and mobile genetic elements in diarrheal cases, highlighting microbiome instability during infection. Importantly, the unexpectedly higher abundance of antibiotic resistance genes in non-diarrheal children underscores the underestimated role of healthy populations as reservoirs of resistance. Furthermore, the establishment of predictive models for diarrhea status and pathogen carriage enhances the translational value of the study. Overall, this work advances the understanding of pediatric diarrheal disease and informs prevention, surveillance, and treatment strategies in the context of global antimicrobial resistance.

## INTRODUCTION

Diarrhea poses a substantial health threat worldwide, especially in developing countries, where it is the second leading cause of death among young children under 5 (1, 2). This condition not only results in a considerable number of deaths but also contributes to growth impairment in many children worldwide, adversely affecting their overall health and well-being (3). Rehydration and maintenance of fluid-electrolyte balance remain the cornerstone of managing diarrheal disease in children (4), whereas antibiotic therapy is reserved for selected cases with suspected or confirmed bacterial etiology (5). Therefore, accurate differentiation between bacterial and non-bacterial etiologies is critical for optimizing antimicrobial use and reducing unnecessary antibiotic exposure (6).

Antimicrobial resistance has emerged as a significant threat in the 21st century because of excessive antimicrobial usages (7). Antibiotic resistance genes (ARGs) can be transferred among different gut bacterial species as well as within the same species, thereby promoting their acquisition and spread (8). Therefore, understanding and mitigating the spread of ARGs are crucial for the effective management of human health (9). Diarrhea is typically caused by pathogenic microbes that utilize their virulence factors (VFs) to infect the gastrointestinal tract, resulting in gastrointestinal symptoms (10). VFs are pivotal to microorganism pathogenicity because they encode functions such as invasion, adhesion, toxin activity, and iron uptake (11). Investigating VFs is essential for understanding the pathogenic mechanisms of diverse microorganisms. Numerous studies have highlighted the importance of examining VFs within the gut microbiota to unravel the complexities of pathogenesis in diarrheal diseases (12, 13).

By disrupting the balance of the gut microbiota, diarrhea can facilitate the colonization and expansion of pathogens, which in turn promotes the transfer of antimicrobial resistance genes (ARGs) and virulence factors (VFs) (14). This gene transfer may involve two conceptually distinct directions of gene flow: pathogens may acquire ARGs from the resident gut microbiota, whereas ARGs or VFs may also be transferred from external pathogens to the resident gut microbiota. Although the latter process may not immediately manifest as clinical symptoms, such as diarrhea, it may create a hidden reservoir capable of continuously acquiring and retaining ARGs and VFs, thereby increasing the difficulty of detection and intervention. Comparative analyses of gut microbiome composition and functionality between children with and without diarrhea can provide valuable insights into the microbiome’s role in the pathogenesis of diarrheal diseases. Such studies are essential for creating effective strategies to prevent and treat these diseases, especially considering the increasing incidence of antimicrobial resistance. However, most previous studies have focused exclusively on either individuals with diarrhea or healthy individuals (15, 16), without conducting comparative analyses between these two groups.

To further investigate the differences in gut microbiota between children with and without diarrhea, as well as the potential transmission pathways of ARGs and VFs, we performed the individually matched case-control study and the metagenomic analyses to annotate, characterize, and compare these features while evaluating their correlations. Specifically, to minimize the potential confounding effects of factors such as age, gender, season of illness onset, hygiene practices, and nutritional status on the study results, we implemented matched case-control sampling for each participant. By developing a random forest (RF) model, we identified specific intestinal microbiota potentially conferring resistance to infections and potential biomarkers for rapid diagnosis and prevention of diarrhea. This study provides deeper insights into the complex interactions among gut microbiota, VFs, ARGs, and MGEs in the context of diarrhea. These findings offer comprehensive information and valuable outcomes for the development of preventive strategies to manage diarrheal diseases and improve children’s health.

### Highlights

1. Proteobacteria increased and alpha diversity decreased in diarrheal children.
2. ARGs were more diverse and abundant in non-diarrheal children.
3. ARGs and VFs were more correlated with MGEs than with bacterial communities.
4. *Actinomyces* may protect asymptomatic carriers from diarrhea.

## MATERIALS AND METHODS

### Sample collection

A total of 716 fecal samples were collected from 323 diarrheal children and 393 non-diarrheal children, aged between 1 and 5 years. In this study, diarrhea was defined as the passage of more than three loose or watery stools within a 24-h period. Children with diarrhea were brought to the hospital within 24 h after the onset of symptoms, and stool samples were collected on the day of the hospital visit before antibiotic administration. None of the diarrheal or non-diarrheal children enrolled in this study had a history of long-term or recent use of probiotics or prebiotics. All stool samples were collected on the day of the hospital visit and stored at −80°C until further analysis. The inclusion criterion for non-diarrheal children’s samples was a regular defecation pattern, no occurrence of diarrhea, and no history of antibiotic use in the past month. The research protocol was approved by the Ethics Committee of the National Institute for Communicable Disease Control and Prevention of the Chinese Center for Disease Control and Prevention.

In this study, a strict 1:1 matching strategy was implemented to minimize the potential confounding effects of factors between the diarrhea group and the non-diarrhea group. Background information was collected through on-site interviews, and careful balancing was conducted for factors such as gender, season of illness onset, hygiene practices, and nutritional conditions to minimize potential bias. Specifically, to ensure the comparability of the age distribution, for children aged 12–23 months, the age difference between matched pairs was restricted to a maximum of 2 months. For those aged 24–59 months, the age difference was limited to 4 months, while for children aged 60 months and older, the difference was capped at 6 months. Finally, a total of 84 samples were chosen, including 42 fecal samples from 323 children with diarrhea (diarrhea group; Table S1) and 42 fecal samples from 393 non-diarrheal children (non-diarrhea group; Table S1). These stringent pairing methods, by controlling for confounding factors, reducing sampling error, and avoiding population ecological fallacies due to individual variation, render the results of this study more authentic and reliable.

### DNA extraction

DNA extraction was performed using 0.25 g of stool samples with the QIAamp PowerFecal DNA Kit (QIAGEN, USA) in accordance with the manufacturer’s instructions. After measuring the DNA concentration with a NanoDrop 2000 UV-Vis Spectrophotometer (Thermo Fisher, USA), the extracted DNA samples were stored at −80°C.

### Metagenomic sequencing and quality control

The MGIEasy FS DNA Library Prep Set was utilized for the construction of DNA libraries in this study. After library preparation, precise quantification was conducted using the Agilent 2100 instrument. High-throughput sequencing was conducted using the MGISEQ-2000RS platforms (MGI, China). To maintain the integrity of the sequencing data, a comprehensive set of quality control procedures was applied to the raw reads. These measures involved eliminating sequences contaminated with adapters, excluding reads containing ambiguous or low-quality bases, and retaining reads with at least 60% high-quality bases. To obtain clean sequencing reads, Bowtie2 (version 2.5.0) was used to align the raw sequencing data to the human reference genome (GRCh38). Reads aligned to the host genome were removed to retain only non-host (microbial) sequences for subsequent analysis. The basic sequencing information is provided in Table S2. The resulting clean reads were then aligned to bacterial genomes from the National Center for Biotechnology Information GenBank using SOAPaligner (version 2.21). The raw sequence data of metagenomes reported in this paper have been deposited in the Sequence Read Archive (SRA) under the BioProject PRJNA1221657.

Microorganism identification was conducted using the method of Kraken (17) and the PFI (MGI Pathogeny Fast Identification pipeline) (18). In addition, Bowtie2 (19) and SAMtools (20) were used to extract specific, targeted reads from the metagenomic data.

### Annotation of ARGs, MGEs, and VFs and pathogen detection

The ARGs-OAP v2.0 analysis pipeline was utilized for the annotation of ARGs (21). The annotation and abundance calculation of MGEs were performed using the Mobile Genetic Element Database (22). VFs were annotated by performing a BLAST search against the VF database (VFDB), applying an identity threshold of 70% and a coverage threshold of 90% (23). Moreover, 16S rRNA gene sequences were identified using the local BLAST algorithm with the Greengenes database as a reference (24). The abundance of 16S sequences in each sample was assessed using Bowtie2 (19) and SAMtools (20).

The abundance of each ARG, MGE, and VF subtype was normalized by the 16S rRNA gene abundance in the same sample and expressed as copies per 16S rRNA gene. Total ARG, MGE, and VF abundance was calculated as the sum of the 16S-normalized abundances of all detected subtypes within each corresponding category.

### Statistical analysis

Principal coordinate analysis (PCoA) was employed to decrease data dimensionality and visualize the relationships among samples. The unweighted pair group method with arithmetic mean (UPGMA) was used to group similar samples based on diversity indices. Alpha diversity indices, including Shannon and Simpson, were calculated using QIIME (25). The Kruskal-Wallis test was used to determine significant differences between non-diarrheal and diarrheal groups. Differential abundance analysis between the groups was performed using statistical analysis of metagenomic profiles (STAMP).

The predominance of vertical versus horizontal transmission of ARGs and VFs was compared using bidirectional modeling and prediction, which were conducted with two-way orthogonal partial least squares (O2PLS) via the R package OmicsPLS (version 2.02) (26).

LEfSe was used to identify genus-level discriminative taxa between the diarrheal and non-diarrhea groups and, separately, between diarrheal and non-diarrhea subgroups carrying bacterial pathogens. The LEfSe analyses were performed after excluding detected pathogens according to the criteria established by the National Health Commission of the People’s Republic of China (27).

The random forest classifier was constructed using the randomForest package (version 4.6) in R (28). The test data set was composed of microbial annotation results from the diarrhea and non-diarrhea groups. The sequential forward selection method was used to choose the optimal genus combination as features. A fivefold cross-validation procedure was implemented to assess the model, which was repeated 20 times.

## RESULTS

### Composition of bacterial communities in diarrheal and non-diarrheal children

**Fig 1:**
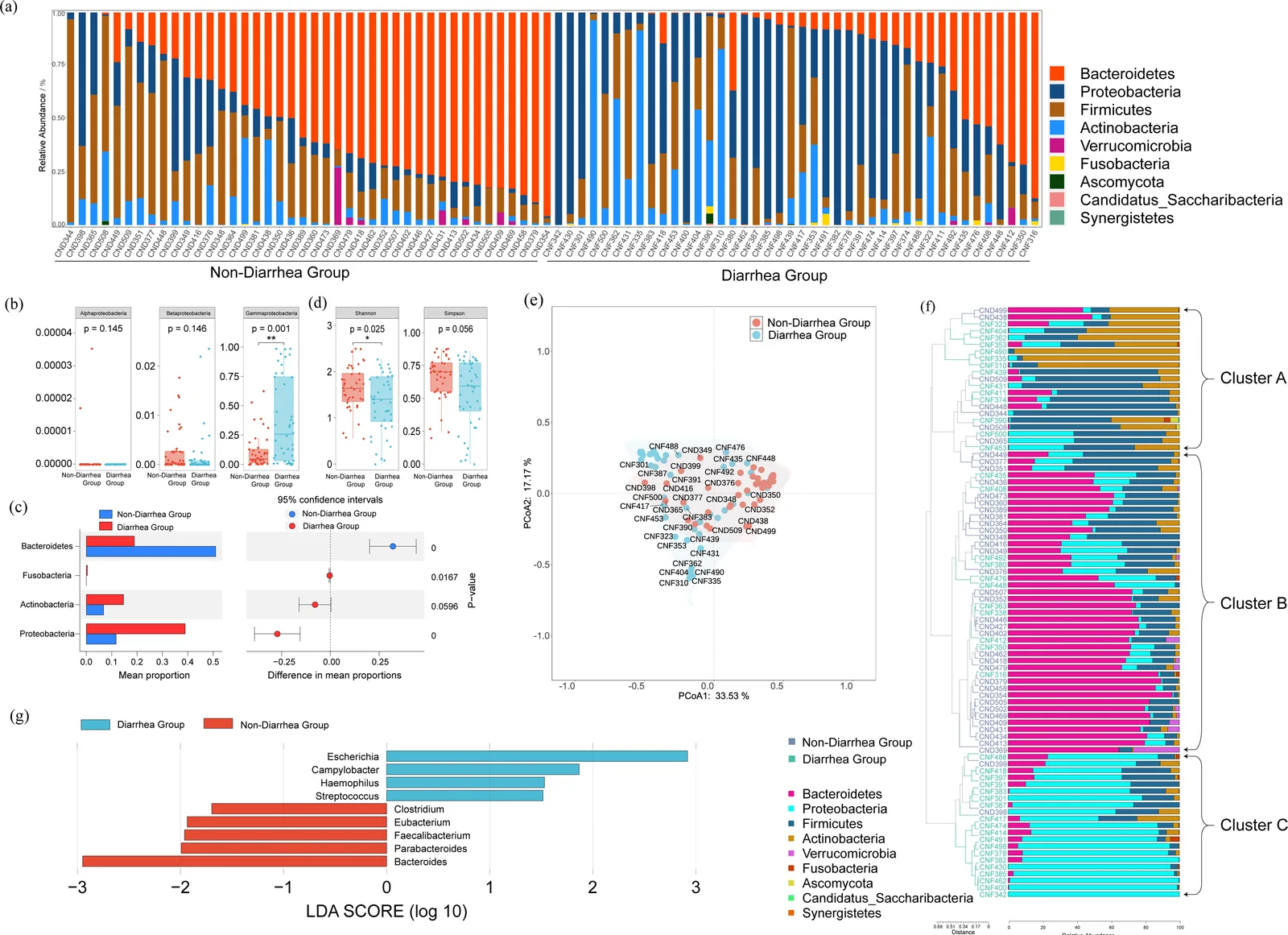
Composition of bacterial communities in the non-diarrhea group and diarrhea group. (**a**) Relative abundance of microbial communities at the phylum level in the non-diarrhea group vs diarrhea group. (**b**) Comparative relative abundance of α-, β-, and γ-*Proteobacteria* in the non-diarrhea group and diarrhea group. (**c**) Comparison of *Actinobacteria*, *Bacteroidetes*, *Fusobacteria*, and *Proteobacteria* abundances based on STAMP analysis. (**d**) Comparison of alpha diversity analyses between the diarrhea group and non-diarrhea group. (**e**) PCoA of all samples. (**f**) UPGMA analysis of all samples with clustering into three groups based on microbial abundance and composition similarity. (**g**) LEfSe analysis was performed at the genus level across the entire microbial community.

Bacterial community structures of stool samples from the diarrheal and non-diarrheal children were categorized into nine phyla, 18 classes, 34 orders, 74 families, and 175 genera (Table S3). Bacteroidetes (52.4%) was the predominant phylum in the non-diarrhea group, whereas Proteobacteria (41.4%) showed the highest overall abundance in the diarrhea group (Fig. 1a). However, individual-level profiles revealed substantial heterogeneity within the diarrhea group. Specifically, not all diarrheal samples were dominated by Proteobacteria; instead, Bacteroides and Actinobacteria predominated in the gut microbiota of seven and five patients, respectively. To investigate the role of Proteobacteria, the primary phylum encompassing most pathogenic and opportunistic bacteria (29), a comparative analysis of its abundance between the diarrhea group and the non-diarrhea group was performed, with class-level differences among *Alphaproteobacteria*, *Betaproteobacteria*, and *Gammaproteobacteria* assessed using Kruskal-Wallis tests.

The results demonstrated that the abundance of *Alphaproteobacteria* and *Betaproteobacteria* showed no significant differences between the diarrheal and non-diarrhea groups (Kruskal-Wallis test, *P* > 0.05; Fig. 1b). In contrast, a significant difference in abundance was observed for *Gammaproteobacteria* between the diarrhea group and non-diarrhea group (Kruskal-Wallis test, *P* < 0.05; Fig. 1b). Subsequent analysis indicated that the predominant genera within the *Gammaproteobacteria* class in the diarrhea group included *Escherichia*, *Salmonella*, *Actinobacillus*, and *Aggregatibacter* (Kruskal-Wallis test, *P* < 0.05; Fig. S1a).

STAMP analysis validated the differential abundance, revealing that *Bacteroidetes* were significantly more abundant in the non-diarrhea group, whereas *Proteobacteria* and *Actinobacteria* were more abundant in the diarrhea group (Fig. 1c). *Bacteroidia* (at class level), including *Bacteroides*, *Parabacteroides*, and *Alistipes,* were significantly more abundant in the non-diarrhea group, whereas *Escherichia* and *Haemophilus* in the *Gammaproteobacteria* (at class level) were significantly more abundant in the diarrhea group (Fig. S1b). Furthermore, the *Bacteroidetes*-to-*Firmicutes* ratio (30), an indicator of gut health, was significantly higher in the non-diarrhea group compared to the diarrhea group (Kruskal-Wallis test, *P* < 0.05; Fig. S1c).

Alpha diversity indices (Shannon indices) indicated that the bacterial communities in the non-diarrhea group exhibited significantly higher diversity than those in the diarrhea group (*P* < 0.05; Fig. 1d). PCoA was conducted at the genus level across all samples (Fig. 1e), and the samples clustered into two groups based on the Bray-Curtis diversity distance, representing the non-diarrhea group and diarrhea group, with partial overlap. The non-diarrhea group showed a more concentrated bacterial community structure, whereas the diarrhea group displayed increased dispersion. There were clear differences in bacterial composition between these two clusters.

In this study, UPGMA was used to classify bacterial communities by clustering samples with similar composition and abundance (Fig. 1f). The diarrhea group was clearly divided into two distinct clusters: clusters A and C. Cluster A was characterized by a predominance of *Actinobacteria* (41.02%) and *Firmicutes* (39.89%). This cluster also included substantial proportions of *Proteobacteria* (12.46%) and *Bacteroidetes* (5.91%). Cluster C was marked by an overwhelming prevalence of *Proteobacteria* (79.30%), followed by *Firmicutes* (10.36%), *Bacteroidetes* (6.75%), and *Actinobacteria* (3.04%). In contrast, the samples from the non-diarrhea group were predominantly grouped in cluster B, comprising *Bacteroidetes* (62.25%), *Firmicutes* (22.42%), *Proteobacteria* (9.74%), and *Actinobacteria* (4.00%). These clusters were used to characterize interindividual heterogeneity within the diarrhea group and to provide a basis for subsequent subgroup-level comparisons. At the genus level, *Escherichia* and *Bacteroides* were identified as biomarkers of the diarrhea group and non-diarrhea group, respectively.

PCoA based on all genus-level taxa within the phyla Actinobacteria, Bacteroidetes, Firmicutes, and Proteobacteria (Fig. S2a through d) showed that the non-diarrhea and diarrhea groups were separated only when Actinobacteria- and Proteobacteria-associated genera were analyzed, whereas no clear separation was observed for Bacteroidetes- or Firmicutes-associated genera. These results suggest that the microbial differences between the two groups were more strongly reflected by genus-level compositional variation within Actinobacteria and Proteobacteria. Based on the UPGMA clustering results, PCoA was further performed at the genus level for diarrheal samples within cluster A and cluster C (Fig. S1d). There were clear differences in bacterial composition between these two clusters. However, the diarrheal samples formed two distinct clusters, with no apparent differences in age or season of onset between samples from the two clusters. Further analysis of cluster A identified *Clostridium* and *Neisseria* as the key biomarkers for the non-diarrhea group and diarrhea group, respectively (Fig. S2f).

### Diversity and abundance of VFs in diarrheal and non-diarrheal children

In this study, a total of 13 categories of VFs were identified, including those related to adherence, effector delivery systems, invasion, motility, and others. Overall, 257 genes associated with virulence were detected (Table S4). The primary subtypes of VFs identified in both the non-diarrhea group and diarrhea group included type I fimbriae, associated with bacterial adherence; yersiniabactin, linked to nutritional and metabolic functions; and the T6SS, responsible for effector delivery.

**Fig 2:**
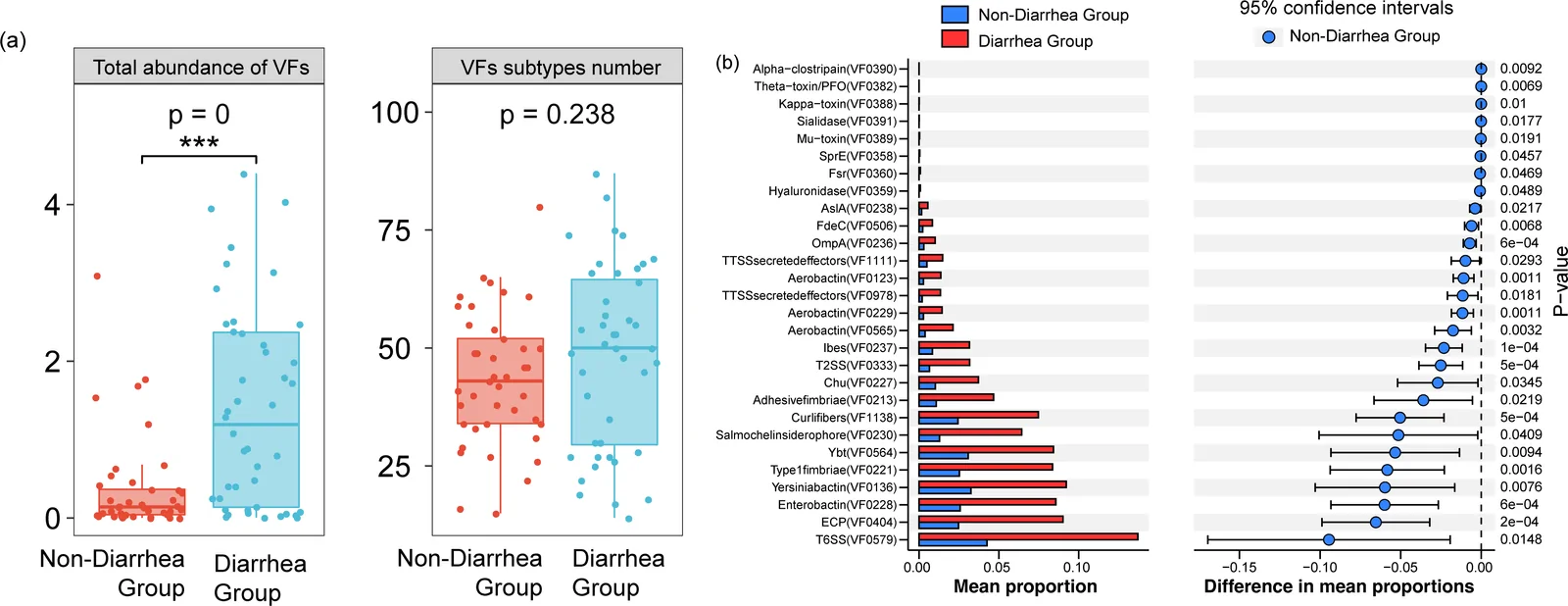
Comparison of abundance and diversity of virulence factors (VFs) between the non-diarrhea group and diarrhea group. (**a**) Comparison of total relative abundance and diversity of VFs between the non-diarrhea group and diarrhea group. (**b**) Comparison of relative abundance of primary VF subtypes between the non-diarrhea group and diarrhea group.

The abundance of VFs was significantly higher in the diarrhea group compared to the non-diarrhea group; however, there were no significant differences in the number of VF subtypes between the two groups (Kruskal-Wallis test, *P* > 0.05; Fig. 2a). In the diarrhea group, VFs associated with nutrition and metabolism, adherence, effector delivery systems, invasion, and motility exhibited a significantly higher abundance compared to the non-diarrhea group (Kruskal-Wallis test, *P* < 0.05; Fig. S3). Multiple subtypes in the diarrhea group, such as T6SS, excretion of cytoplasmic proteins (ECP), enterobactin, yersiniabactin, and type 1 fimbriae, were significantly more abundant than those in the non-diarrhea group (*P* < 0.05; Fig. 2b), indicating an increased prevalence of these factors associated with diarrheal symptoms. Additionally, no significant differences in the abundance and diversity of VFs were detected between cluster A and cluster C of the diarrhea group.

### Diversity and abundance of ARGs in diarrheal and non-diarrheal children

**Fig 3:**
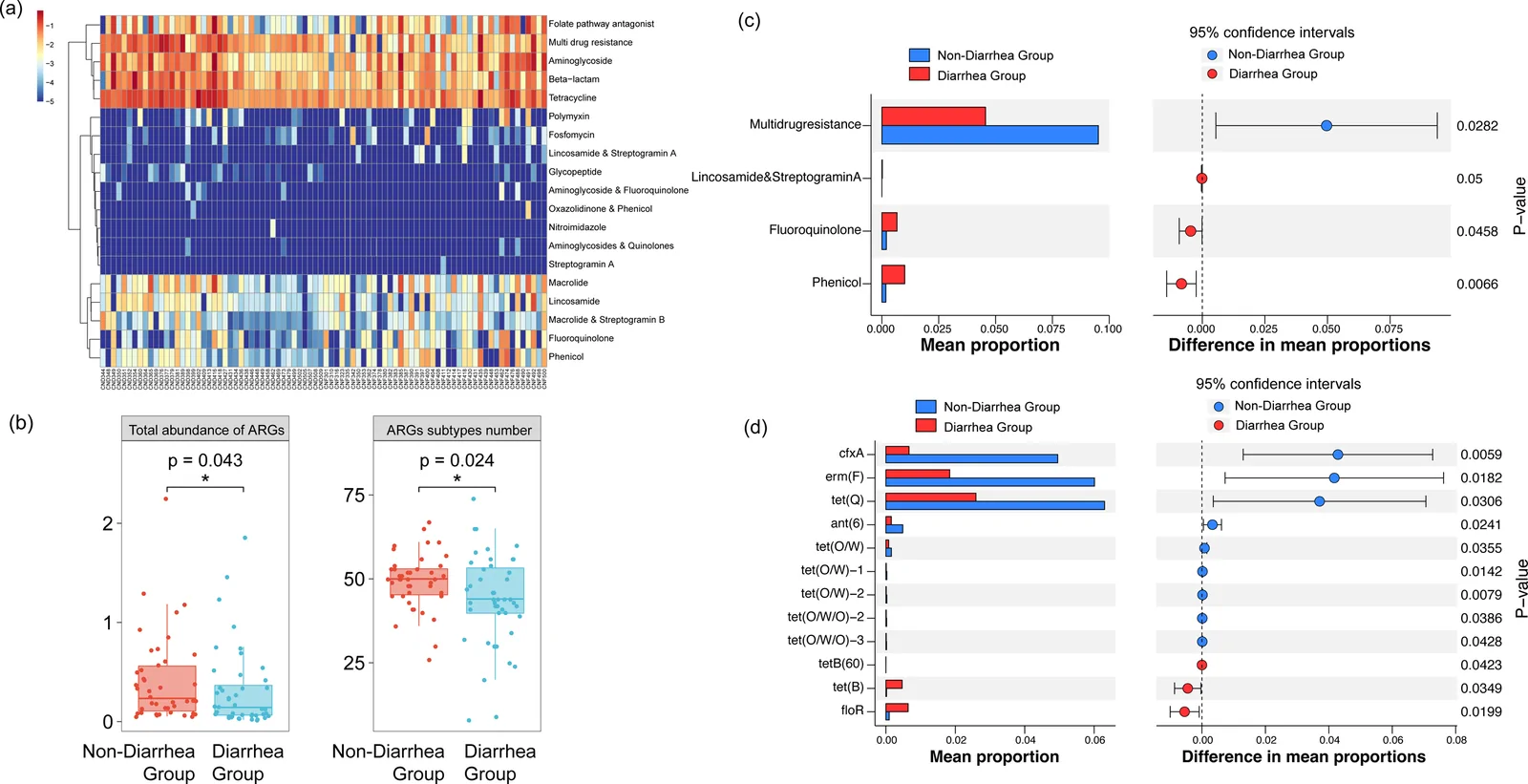
Analysis of relative abundance and diversity of antibiotic resistance genes (ARGs) in the non-diarrhea group and diarrhea group. (**a**) Heatmap of relative abundance of ARGs at the type level across all samples. (**b**) Comparison of total relative abundance and diversity of ARGs between the non-diarrhea group and diarrhea group. (**c** and **d**) Comparison of major ARGs (at type and subtype levels) between the non-diarrhea group and diarrhea group.

In total, 22 types and 154 subtypes of ARGs were detected. Each sample contained between eight and 74 distinct ARG subtypes (Table S5). The most abundant ARGs included tetracycline resistance genes and aminoglycoside resistance genes, followed by multidrug resistance genes and β-lactam resistance genes. Tetracycline, aminoglycoside, multidrug resistance genes, and β-lactam were prevalent categories of ARGs, showing the largest number of subtypes and highest overall abundance in both the non-diarrhea group and diarrhea group (Fig. 3a). The non-diarrhea group showed both a significantly greater diversity of ARG subtypes and a higher total abundance of ARGs compared to the diarrhea group (Kruskal-Wallis test, *P* < 0.05; Fig. 3b).

In the non-diarrhea group, multidrug resistance at the type level was more prevalent, and specific subtypes such as *cfxA*, *erm*(*F*), *tet*(*Q*), *ant*(*6*), and *tet*(*O/W*) were also significantly more prevalent compared to the diarrhea group. Resistance to phenicol and fluoroquinolone was significantly higher in the diarrhea group (Kruskal-Wallis test, *P* < 0.05; Fig. 3c), whereas the *floR* and *tet*(*B*) subtypes were notably more common in the diarrhea group (Kruskal-Wallis test, *P* < 0.05; Fig. 3d).

### Diversity and abundance of MGEs in diarrheal and non-diarrheal children

In this investigation, a total of four types and 37 subtypes of MGEs were identified. Each sample displayed between six and 26 different subtypes of MGEs (Table S6). Specifically, the primary types of MGEs were plasmids (diarrhea: 231, 49.57%; non-diarrhea: 235, 50.43%) and insertion sequences (ISs) (diarrhea: 175, 51.17%; non-diarrhea: 167, 48.83%).

**Fig 4:**
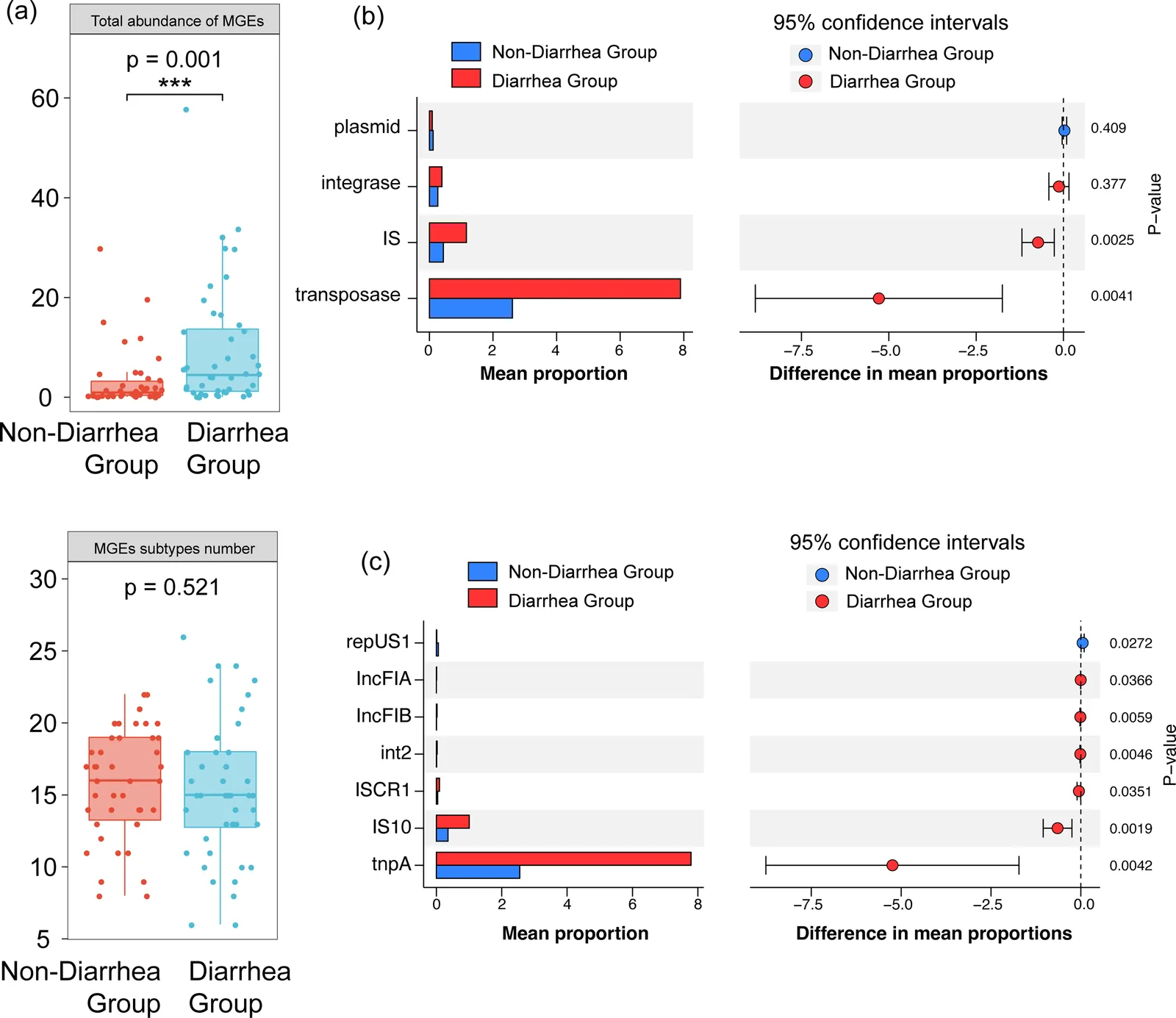
Analysis of relative abundance and diversity of mobile genetic elements (MGEs) in the non-diarrhea group and diarrhea group. (**a**) Comparison of total relative abundance and diversity of MGEs between the non-diarrhea group and diarrhea group. (**b** and **c**) Comparison of major MGEs (at type and subtype levels) between the non-diarrhea group and diarrhea group.

There were no significant differences in the number of MGE subtypes between the non-diarrhea group and the diarrhea group (Kruskal-Wallis test, *P* > 0.05; Fig. 4a), and despite the remarkable diversity of plasmids with numerous subtypes, their total abundance remained relatively low (Fig. S4).

Additionally, the overall abundance of MGEs was significantly higher in the diarrhea group than in the non-diarrhea group (Kruskal-Wallis test, *P* < 0.05; Fig. 4a). This remarkable difference was principally attributed to the increased abundance of transposase and ISs in the diarrhea group (Kruskal-Wallis test, *P* < 0.05; Fig. 4b). The *tnpA* and *IS10* were significantly more abundant in the diarrhea group than in the non-diarrhea group (*P* < 0.05; Fig. 4c), with the *tnpA* contributing 98.3% of the transposase abundance. In contrast, plasmid-associated MGEs showed greater subtype diversity and relatively higher abundance in the non-diarrheal group, mainly represented by *repUS1* and *rep1*. Among integrase-related elements, *intI1* was the dominant subtype and was more abundant in diarrheal samples.

The O2PLS method was utilized to analyze the data matrices from the non-diarrhea group and diarrhea group, focusing on bacterial community (BAC, at the phylum-level abundance), MGEs (at the type-level abundance), and ARGs (at the type-level abundance). Correlations were calculated, and potential routes of transfer and dissemination were presumed (Table 1). The variation in the data sets (ARGs-BACs, ARGs-MGEs, VFs-BACs, and VFs-MGEs) was decomposed by the O2PLS approach into three components: a common segment reflecting shared variation, an orthogonal segment showing variation unique to each group, and a noise segment that captures redundant information. To refine the model, several pre-establishments and cross-validations were conducted. This process allowed for the identification of the minimum prediction error and the optimal number of components for both the joint and orthogonal parts. These components relate to X, representing the abundance of ARGs and VFs, and Y, encompassing the abundance of the BAC and MGEs. In the optimized models identified, the variation in the joint parts was analyzed and compared in parallel.

**TABLE 1:** The contributions of bacterial communities and MGEs in the orthogonal and correlated components

Group	Data set	R2X	R2Y	R2Xcorr	R2Ycorr
Non-diarrhea group	ARG-BAC	0.559	0.714	0.327	0.433
	ARG-MGE	0.374	0.954	0.237	0.886
	VF-BAC	0.671	0.611	0.671	0.611
	VF-MGE	0.632	0.967	0.439	0.948
Diarrhea group	ARG-BAC	0.718	0.651	0.34	0.477
	ARG-MGE	0.479	0.892	0.237	0.821
	VF-BAC	0.434	0.457	0.434	0.457
	VF-MGE	0.597	0.804	0.257	0.804

The contributions of MGEs and BACs to the variation in ARGs and VFs, respectively, were further determined (*R*^2^Ycorr). Overall, in both the non-diarrhea group and diarrhea group, MGEs contributed more to the variation in ARGs and VFs compared to BACs. This pattern indicates that ARG and VF profiles were more strongly associated with MGE profiles than with broad bacterial community composition. These findings are consistent with the potential role of MGE-linked processes in shaping ARG/VF variability. Notably, in comparison to the diarrhea group, the ARGs and VFs in the non-diarrhea group samples showed a stronger correlation with MGEs.

### Comparison of diarrheal and non-diarrheal children carrying bacterial pathogens

Healthy individuals carrying pathogens without showing clinical symptoms is a phenomenon worth studying; however, the specific reasons still lack concrete evidence. Therefore, 12 common diarrhea-associated pathogens, including bacterial, viral, and protozoal targets, were screened in both the non-diarrhea and diarrhea groups using PCR (Fig. S5), following established experimental protocols. The screening panel included *Salmonella*, five categories of pathogenic *Escherichia coli* (EPEC, ETEC, EIEC, EHEC, and EAEC), *Vibrio parahaemolyticus*, *Yersinia enterocolitica*, *Clostridium difficile*, *Giardia lamblia*, adenovirus, and norovirus.

Because the subsequent subgroup analysis was intended to evaluate bacterial pathogen carriage, only samples positive for bacterial pathogens were included in this comparison. The B-diarrheal subgroup was defined as diarrheal samples positive for at least one bacterial pathogen, whereas the B-non-diarrheal subgroup was defined as non-diarrheal samples positive for at least one bacterial pathogen. This analysis was performed to explore microbial features associated with symptomatic versus asymptomatic bacterial pathogen carriage. EHEC type *Escherichia coli* was not detected in all samples.

**Fig 5:**
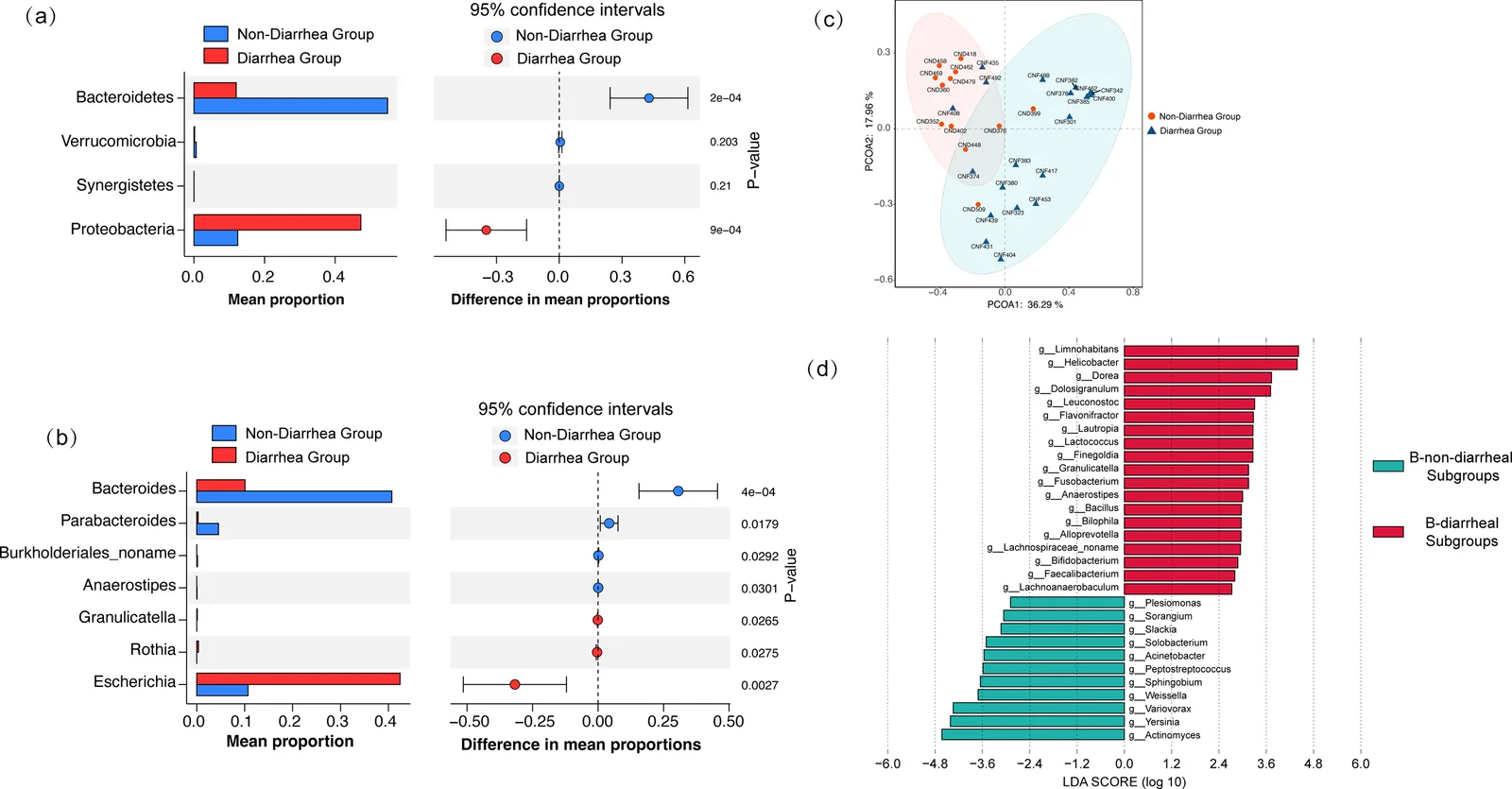
Analysis of bacterial communities in pathogen‑positive diarrheal and non‑diarrheal samples. (**a** and **b**) Comparison of major bacteria (at the phylum and genus level) between the non-diarrhea group and diarrhea group. (**c**) PCoA of samples positive for pathogenic. (**d**) Biomarkers were separately screened from the B-diarrheal subgroup and B-non-diarrheal subgroup based on LEfSe analysis.

In the study, 12 non-diarrhea group samples and 20 diarrhea group samples tested positive for bacterial pathogens, forming the B-non-diarrheal and B-diarrheal subgroups, respectively. At the phylum level, *Bacteroidetes* was significantly more abundant in the B-non-diarrheal subgroup, whereas *Proteobacteria* exhibited markedly higher abundance in the B-diarrheal subgroup (Fig. 5a). At the genus level, *Bacteroides* and *Parabacteroides* exhibited significant enrichment in the B-non-diarrheal subgroup, while *Escherichia* was notably more prevalent in the B-diarrheal subgroup (Fig. 5b). PCoA further emphasized the distinct differences in bacterial composition between the B-non-diarrheal and B-diarrheal subgroups (Fig. 5c).

To identify genus-level biomarkers associated with symptomatic versus asymptomatic bacterial pathogen carriage, LEfSe analysis was further performed in the B-diarrheal and B-non-diarrheal subgroups (Fig. 5d). The results revealed that *Actinomyces* were the most prominent biomarkers in the B-non-diarrheal subgroup, while *Limnohabitans* and *Helicobacter* were the most prominent biomarkers in the B-diarrheal subgroup.

### Predicting pathogen carriage in diarrheal children with random forest modeling

The variety of pathogenic microorganisms that can cause diarrhea is extensive, yet current clinical detection typically targets only a few common pathogens such as *Salmonella* and *Shigella*. Furthermore, these detection methods usually involve a lengthy process of microbial culturing, making it difficult to accurately identify the specific diarrheal pathogens, and as a result, targeted antibiotic therapy is often not feasible. However, the widespread use of antibiotics to treat diarrhea can easily lead to dysbiosis of the intestinal microbiota and may increase the risk of gut microbes acquiring ARGs. Therefore, the fast determination of whether to use antibiotics in patient treatment carries clinical importance.

**Fig 6:**
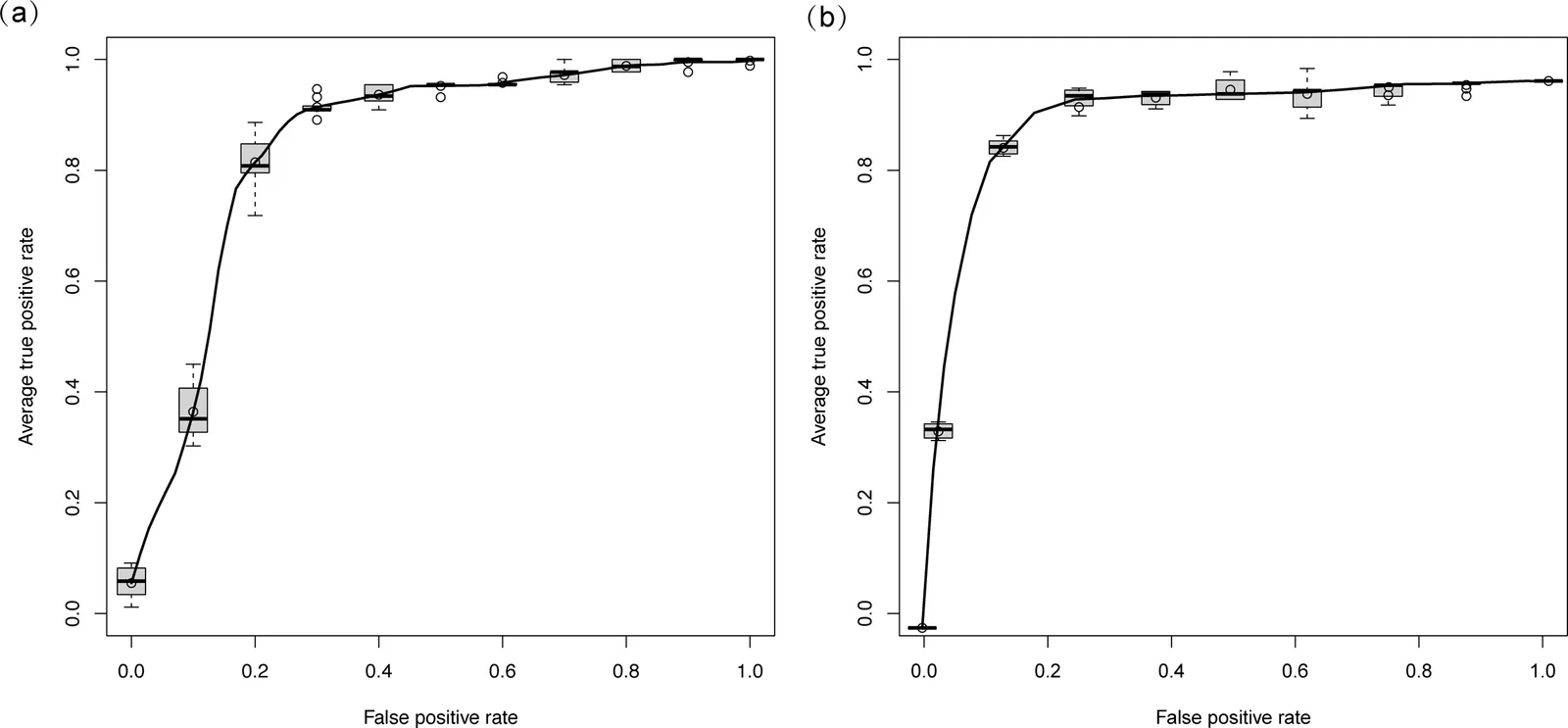
The model for distinguishing between bacterial diarrhea and nonbacterial diarrhea states (**a**) and predicting the presence of pathogenic microorganisms (**b**) was constructed using the random forest and LEfSe methods.

To predict bacterial diarrhea versus non-bacterial diarrhea, a random forest (RF) model was constructed. The RF model revealed strong predictive accuracy on a test data set, with an accuracy of 82.62%, recall of 85.68%, specificity of 85.23%, and an area under the curve (AUC) of 0.8485 (Fig. 6a). These metrics suggest that the RF model may help distinguish bacterial diarrhea from nonbacterial diarrhea, highlighting the potential use of these nonpathogenic bacterial combinations as predictive microbial features.

An LEfSe-based feature selection strategy identified *Megasphaera* and *Parasutterella* as key predictive features for pathogenic bacterial carriage in individuals with diarrhea. An RF model constructed using these features achieved an accuracy of 89.09%, recall of 90%, specificity of 88.10%, and AUC of 0.8788 on the test set (Fig. 6b), suggesting their potential value as microbial indicators of pathogen carriage in diarrheal patients.

## DISCUSSION

In this study, groups of children with diarrhea and non-diarrheal children were first established with a strict individually matched case-control study, and their gut microbiota ARGs, VFs, and MGEs were compared and analyzed based on metagenomic sequencing data. Subsequently, the reasons why healthy children carry pathogens without exhibiting clinical symptoms were further investigated. Finally, prediction models for rapidly determining bacterial diarrhea and nonbacterial diarrhea status, as well as the presence of pathogens, were constructed using the Random Forest method.

In the diarrhea group, an increased abundance of *Proteobacteria* was observed along with a more intricate distribution of bacterial communities. In contrast, the non-diarrhea group exhibited higher alpha diversity and displayed a higher prevalence of core gut commensal bacteria, including *Bacteroides*, *Parabacteroides*, and *Alistipes*, in the phylum *Bacteroidetes*. In addition, the diarrhea group exhibited a lower abundance of ARGs, accompanied by higher levels of MGEs and VFs.

In agreement with prior studies, the analysis revealed that *Bacteroides* and *Firmicutes* were the primary bacterial phyla in the gastrointestinal tract in the non-diarrhea group (31). However, the gut microbiota of the diarrhea group was principally composed of *Proteobacteria*, followed by *Firmicutes*, *Bacteroidetes*, and *Actinobacteria*. The diarrhea group showed decreased gut microbiota diversity, indicating a possible disturbance and imbalance within the gut microbiota (32, 33). Reduced abundance of *Bacteroidetes (*34, 35) and increased presence of *Firmicutes* have also been associated with diarrhea (36–38). Furthermore, a decreased ratio of *Bacteroidetes* to *Firmicutes* may be linked to the occurrence of diarrhea (30). The increased abundance of *Proteobacteria* has been linked to adverse health effects, with certain genera, such as *Escherichia* and *Salmonella*, considered potential agents of diarrhea (39–41).

An additional finding of this study is the marked heterogeneity within the diarrhea group. Although Proteobacteria showed the highest overall abundance in diarrheal children, not all diarrheal samples were dominated by Proteobacteria, and Bacteroides or Actinobacteria remained predominant in a subset of patients. This suggests that diarrhea-associated microbial alterations in this cohort were not uniform but instead may represent multiple ecological configurations. Such heterogeneity raises the possibility that differences in underlying community structure may be associated with distinct microbial shifts during diarrheal episodes. PCoA of all genus-level taxa within the phyla Actinobacteria, Bacteroidetes, Firmicutes, and Proteobacteria indicated that the microbial differences between the non-diarrhea and diarrhea groups were driven primarily by genus-level compositional variation within Actinobacteria and Proteobacteria, rather than within Bacteroidetes or Firmicutes. Moreover, the identification of *Clostridium* and *Neisseria* as the key biomarkers of cluster A in the non-diarrhea and diarrhea groups, respectively, further suggests that the heterogeneity of this cluster is characterized by distinct genus-level microbial signatures.

LEfSe analysis of the bacterial communities in diarrhea and non-diarrhea samples harboring pathogenic organisms identified *Actinomyces* as a putative biomarker associated with the non-diarrheal status. Previous studies have reported that members of the genus Actinomyces can produce various bioactive metabolites with free radical-scavenging activity and inhibitory effects against several pathogens, including methicillin-resistant *Staphylococcus epidermidis* (MRSE) strains (42). These findings suggest that the enrichment of Actinomyces may be associated with asymptomatic bacterial pathogen carriage. Furthermore, *Helicobacter pylori* infection may impair gastric acid barrier function, reduce the elimination of ingested pathogens, and thereby increase susceptibility to secondary infections and diarrhea in bacteria colonized children (43). However, as an environmental microbe, *Limnohabitans* has not been reported to have an association with diarrheal diseases. As a biomarker associated with the pathogen-carrying diarrheal microbial community, *Limnohabitans* deserves further research attention to clarify its relevance and potential role in diarrheal disease.

Within the One Health framework, the intestinal microbiota of healthy individuals is considered a major reservoir for ARGs and serves as a site for the transfer of these genes (44). Hence, the issue of ARG carriage in healthy individuals must receive prioritized attention. This study underscored significant variations in the abundance of ARGs between the two groups, with higher levels of ARGs and lower diversity in ARG subtypes observed in the non-diarrhea group. A healthy gut typically contains functional ARGs, and the prevalence of tetracycline resistance genes, including *tetX*, was observed in both groups. Antibiotic use is correlated with higher levels of *cfxA* and *tetQ* in individuals with diarrhea (45). Therefore, those who had taken antibiotics were excluded from this cohort. The difference in ARG levels between the two groups may be attributed to the cumulative prior use of these antibiotics, indicating that antibiotic use can affect the occurrence of certain ARGs (46). VFs and ARGs can assist bacteria in obtaining superior survival conditions within the host body. However, in the absence of antibiotic threats within the healthy human body, the exchange of VFs and ARGs not only increases the diversity and abundance of the bacteria carrying these elements but may also elevate the potential and frequency of their transfer to pathogens (47). This study found that compared to the diarrhea group, the correlation between ARGs, VFs, and MGEs was stronger in the non-diarrhea group samples. MGEs play a crucial role in the spread of antibiotic resistance genes among pathogens (48), and their prevalence can be stimulated by antibiotic use (49). The prevalence of additional MGE subtypes, such as IS10 (50) and intI1 (51), was also observed, and these have important implications for antibiotic resistance and gene transfer. O2PLS analysis showed that ARG and VF profiles shared stronger covariance with MGE profiles than with phylum-level bacterial community composition. This stronger association is consistent with previous reports suggesting that MGEs may play a more prominent role in gene mobility (52).

Microorganisms employ VFs to forge interactions with their hosts, resulting in severe and potentially life-threatening infections (53–56). In this study, the diarrhea group exhibited a significantly higher abundance of VFs, particularly T6SS, ECP, Enterobactin, Yersiniabactin, and type 1 fimbriae. These VFs are essential for microbial competition and infection (57–60). In contrast, non-diarrheal children with pathogenic infections showed a greater abundance of *Bacteroides*, which synthesize butyrate and promote gastrointestinal health (61). This abundance may enhance their resistance to diarrheal symptoms. However, the diarrhea group with pathogens exhibited a higher abundance of VFs, further amplifying their susceptibility to infection.

To achieve rapid classification of diarrheal disease and prediction of its potential etiology, we constructed microbiome-based predictive models. The predictive performance of our models reached a level comparable to or higher than that of previously reported diarrheal disease prediction models (62, 63), supporting the potential value of nonpathogenic microbial features in etiological differentiation and pathogen-carriage prediction. The biomarker *Megasphaera* (64) has been previously established as a dependable indicator for differentiating between patients with diarrhea and those without, in addition to serving as a reliable predictor of pathogen carriage in individuals. The predictive performance of this model suggests the potential value of these microbial features in supporting microbiome-based differentiation of diarrheal diseases. *Parasutterella* has been identified as one of the core microorganisms in the human intestinal tract and is associated with a variety of health outcomes (65). Building upon previous research, this study identified *Parasutterella* as a predictive feature for distinguishing between bacterial and non-bacterial diarrhea, thereby enhancing the referential value and clinical significance of the diagnostic results.

In conclusion, this study utilized the individually matched case-control study and metagenomic sequencing to examine bacterial communities, VFs, MGEs, and ARGs in the gut microbiota of children with and without diarrhea. Significant differences in the composition and abundance of these elements were observed between the groups. Using the random forest model, we identified specific gut microbial taxa associated with diarrheal status and potential bacterial etiology. Furthermore, our analysis revealed candidate microbial biomarkers that may help support etiological differentiation and inform appropriate antimicrobial decision-making for bacterial diarrheal diseases. These findings provide valuable insights into microbiome features associated with pediatric diarrhea and their potential clinical relevance for gut health management. Additionally, it underscored the importance of monitoring healthy individuals for the transfer of antibiotic resistance genes and the suppression of antimicrobial resistance.

## Supplementary Material

Reviewer comments

## Data Availability

The raw metagenomic data of all samples in this study have been submitted to NCBI under the BioProject accession number PRJNA1221657. A STORMS checklist is available at https://doi.org/10.5281/zenodo.20066338 (66).

## SUPPLEMENTAL MATERIAL

Supplemental Figures msystems.00198-26-s0001.docx Figures S1-S5.

Supplemental Tables msystems.00198-26-s0002.xlsx Tables S1-S6.

OPEN PEER REVIEW reviewer-comments.pdf An accounting of the reviewer comments and feedback.

ASM does not own the copyrights to Supplemental Material that may be linked to, or accessed through, an article. The authors have granted ASM a non-exclusive, world-wide license to publish the Supplemental Material files. Please contact the corresponding author directly for reuse.
