# Supplementary material for: Comparative gut microbiome in diarrheal and non-diarrheal children: an individually matched case-control study
Source: mSystems. 2026 Sep 30;11(10):e00198-26. doi: 10.1128/msystems.00198-26 (PMC13712888; doi:10.1128/msystems.00198-26)
Supplement: Supplementary file 1 [file reviewer-comments.pdf]

ASM | mSystems

Comparative Gut Microbiome in Diarrheal and Non-Diarrheal Children: An Individually Matched Case-Control Study

Xin Lu, Jialiang Xu, Fan Yufeng, Geruo Qu, Jiaqi Li, Yao Peng, Mengyu Wang, Jingyun Zhang, Yuqing Feng, Xiao Liu, Yongfei Hu, Biao Kan, Zhenpeng Li, and Mei Zeng

Corresponding Author(s): Xin Lu, National Institute for Communicable Disease Control and Prevention

Review Timeline:

Submission Date:	February 13, 2026
Editorial Decision:	March 17, 2026
Revision Received:	May 11, 2026
Editorial Decision:	June 4, 2026
Revision Received:	July 1, 2026
Accepted:	August 5, 2026

Editor: Shrikant Mantri

Reviewer(s): Disclosure of reviewer identity is with reference to reviewer comments included in decision letter(s). The following individuals involved in review of your submission have agreed to reveal their identity: Ardhendu Chakrabortty (Reviewer #1); Shuo Zhao (Reviewer #2)

Transaction Report:

(Note: With the exception of the correction of typographical or spelling errors that could be a source of ambiguity, letters and reports are not edited. The original formatting of letters and referee reports may not be reflected in this compilation.)

DOI: https://doi.org/10.1128/msystems.00198-26

Re: mSystems00198-26 (**Comparative Gut Microbiome in Diarrheal and Non-Diarrheal Children: An Individually Matched Case-Control Study**)

Dear Dr. Xin Lu:

Thank you for the privilege of reviewing your work. Below you will find my comments, instructions from the mSystems editorial office, and the reviewer comments.

Please return the manuscript within 60 days; if you cannot complete the modification within this time period, please contact me. If you do not wish to modify the manuscript and prefer to submit it to another journal, notify me immediately so that the manuscript may be formally withdrawn from consideration by mSystems.

Revision Guidelines
To submit your modified manuscript, log into the submission site at https://msystems.msubmit.net/cgi-bin/main.plex. Go to Author Tasks and click the appropriate manuscript title to begin. The information you entered when you first submitted the paper will be displayed; update this as necessary. Note the following requirements:

• Upload point-by-point responses to the issues raised by the reviewers in a file named "Response to Reviewers," NOT in your cover letter.
• Upload a compare copy of the manuscript (without figures) as a "Marked-Up Manuscript" file.
• Upload a clean .DOC/.DOCX version of the revised manuscript and remove the previous version.
• Each figure must be uploaded as a separate, editable, high-resolution file (TIFF or EPS preferred), and any multipanel figures must be assembled into one file.
• Any supplemental material intended for posting by ASM should be uploaded with their legends separate from the main manuscript. You can combine all supplemental material into one file (preferred) or split it into a maximum of 10 files with all associated legends included.

For complete guidelines on revision requirements, see our Submission and Review Process webpage. Submission of a paper that does not conform to guidelines may delay acceptance of your manuscript.

Data availability: ASM policy requires that data be available to the public upon online posting of the article, so please verify all links to sequence records, if present, and make sure that each number retrieves the full record of the data. If a new accession number is not linked or a link is broken, provide mSystems production staff with the correct URL for the record. If the accession numbers for new data are not publicly accessible before the expected online posting of the article, publication may be delayed; please contact production staff (mSystems@asmusa.org) immediately with the expected release date.

Publication Fees: For information on publication fees and which article types are subject to charges, visit our website. If your manuscript is accepted for publication and any fees apply, you will be contacted separately about payment during the production process; please follow the instructions in that e-mail. Arrangements for payment must be made before your article is published.

ASM Membership: Corresponding authors may join or renew ASM membership to obtain discounts on publication fees. Need to upgrade your membership level? Please contact Customer Service at Service@asmusa.org.

The ASM Journals program strives for constant improvement in our submission and publication process. Please tell us how we can improve your experience by taking this quick Author Survey.

Thank you for submitting your paper to mSystems.

Sincerely,
Shrikant Mantri
Editor
mSystems

Reviewer #1 (Comments for the Author):

Major

This study lacks focus on key aspects, such as how many days after diarrhea the stool sample was collected.
Adjustment of several confounding factors found to be missing, such as diet, nutritional condition, hygiene practice, and

seasonal onset. Health hygiene and nutritional status become significantly important as the healthy individuals also carry pathogenic bacteria.
Interpretations made for the microbiome analysis were mostly generic and already well known, where the article lacks a novelty factor. Looking at the data at the individual level would have provided more novel insights. In Figure 1(a), the author misses to highlight that among the diarrhea group, not only was proteobacteria abundant, but also bacteroides was most abundant in at least 8 subjects and actinobacteria in 5 subjects, which could be because of their starting community, and that could potentially dictate the direction of community shift.
More interesting observations could be drawn if the PCoA analysis were done on taxa not only with the sample.
To target factors preventing disease, LEfSe should be done on the entire community.

Minor
Figure S4: Samples can be clustered, as it is difficult to interpret.
Biomarker analysis is packed with multiple concepts that could have been simplified.
The accuracy and specificity of the random forest model should be compared with available random forest models.
The RF model predicting pathogenic bacteria was low on accuracy.

Reviewer #2 (Comments for the Author):

This study is based on a rigorously matched case-control design and employs metagenomic sequencing to compare the gut microbial composition, ARGs, MGEs, and VFs between diarrheal and non-diarrheal children. The overall research design is well-conceived, and the sample-matching strategy is commendable. The overall research design is reasonable, the sample matching strategy is worthy of recognition, and it has clinical guidance for children with diarrhea. The following are some comments and questions regarding this study.

1. In lines 48-49 of the abstract, due to the lack of specific mechanism research in the study, conclusions regarding asymptomatic carriers should be inferred more cautiously.
2 In Lines 166-169, the description of normalization for ARGs, MGEs, and VFs is unclear.
3. In lines 266-270, the numbers and proportions related to proportions should be explained more clearly.
4. In Section 3.5, since O2PLS can only be used for correlation analysis, the relevant statements regarding the inference of transmission pathways should be more cautious.
5. In Section 3.6, the authors detected three non-bacterial pathogens. However, the use of the group name "bacterial pathogens with diarrhea symptoms (B-diarrheal subgroup)" in lines 337-338 may be confusing.
6. In lines 407-413, since the supporting evidence is based on correlation analysis, the inferences regarding the protective capacity of Actinobacillus and Actinomyces for the host should be stated more cautiously.
9. The description of the research plan for Limnohabitans in lines 416-419 is inappropriate.

Reviewer #3 (Comments for the Author):

This work leverages a carefully matched case-control cohort and whole-metagenome profiling to characterize differences in gut microbiota structure and key functional gene repertoires (including antibiotic resistance determinants, mobile genetic elements, and virulence-associated factors) between children with diarrhea and non-diarrheal counterparts. Overall, the study is thoughtfully organized, and the matching strategy strengthens the comparability of the groups and supports clinically meaningful interpretation for pediatric diarrhea. The following comments and questions are provided to help improve clarity, consistency, and presentation.

1. In line 27, should it be 'Highlights' (possibly a typo)? Also, in the third bullet point, the sentence is missing a period at the end.

2. In the Abstract (lines 33-38), the objective states "determine whether diarrhea is caused by bacterial infection," but the later description indicates a microbiome-based RF classifier. Please clarify in the Abstract whether the model predicts bacterial etiology defined by pathogen testing, or predicts diarrheal status/likelihood based on microbiome features.

3. In the Introduction (lines 58-64), the statement "The primary treatment for severe acute diarrhea episodes is still antibiotic therapy" is overgeneralized. Please revise to a more cautious, guideline-consistent wording.

4. In the Introduction (lines 77-84), "overt" and "covert" gene exchange are introduced as a key conceptual framework. Please add a brief definition or rephrase using standard terminology to avoid ambiguity.

5. In Section 3.1 (lines 231-245), Cluster A/C are introduced with detailed phylum compositions, but their role in the narrative is not clearly stated at this point.

6. In Section 3.2 (lines 253-264), "overall relative abundance" is used as the main descriptor, while Methods state that statistical evaluations are based on "normalized abundance values."

7. In the Conclusion (lines 469-474), phrases such as "may play a crucial role in enhancing the probability of diarrhea" read as causal. Please revise to association-level language consistent with the evidence.

8. In Figure captions, wording such as "samples positive for pathogenic" is grammatically incomplete. Please revise to "samples positive for pathogens/pathogenic microorganisms" and keep the caption language consistent with Section 3.6 terminology.

Comments and suggestions

Major

1. **This study lacks focus on key aspects, such as how many days after diarrhea the stool sample was collected.**
2. **Adjustment of several confounding factors found to be missing, such as diet, nutritional condition, hygiene practice, and seasonal onset. Health hygiene and nutritional status become significantly important as the healthy individuals also carry pathogenic bacteria.**
3. **Interpretations made for the microbiome analysis were mostly generic and already well known, where the article lacks a novelty factor. Looking at the data at the individual level would have provided more novel insights. In Figure 1(a), the author misses to highlight that among the diarrhea group, not only was proteobacteria abundant, but also bacteroides was most abundant in at least 8 subjects and actinobacteria in 5 subjects, which could be because of their starting community, and that could potentially dictate the direction of community shift.**
4. **More interesting observations could be drawn if the PCoA analysis were done on taxa not only with the sample.**
5. **To target factors preventing disease, LEfSe should be done on the entire community.**

Minor

1. **Figure S4: Samples can be clustered, as it is difficult to interpret.**
2. **Biomarker analysis is packed with multiple concepts that could have been simplified.**
3. **The accuracy and specificity of the random forest model should be compared with available random forest models.**
4. **The RF model predicting pathogenic bacteria was low on accuracy.**

Remark to the editor

The manuscript covers multiple important aspects, including microbiome profiling, antimicrobial resistance, virulence analysis and role of mobile genomic elements. Additionally incorporation of machine learninnng based approach to predict biomarkers in diarrheal patient enhances the interdisciplinary appeal. The broad integrative approach makes it relevant for wide range of audience but same time it falls short in novelity factor, as several components appear incremental

rather than conceptually innovative. In certain areas, specially in the microbiome analysis, the findings demands in depth analysis to strengthen the biological insights. A more focused analysis and deeper exploration with key findings will substantially improve the work to better align with the expectations of this journal.

Reviewer #1
Major

1. How many days after diarrhea the stool sample was collected?
Thank you for this important comment. We reviewed the clinical records of the children enrolled in this study cohort and confirmed that all diarrheal cases sought medical care within 1–2 days of symptom onset. All fecal samples were collected on the day of the hospital visit and stored at −80°C until further analysis. This information has been added to the revised manuscript (Lines 132–133).

2. Looking at the data at the individual level would have provided more novel insights.
Thank you for this insightful comment. We agree that examining the data at the individual level provides important information on inter-individual heterogeneity within the diarrhea group. As you pointed out, although Proteobacteria was increased overall in the diarrhea group, not all diarrheal samples were dominated by Proteobacteria. This observation is also consistent with our clustering analysis shown in Figure 1f, in which the diarrhea group could be further divided into distinct subclusters with different phylum-level compositions. These findings support the possibility that the initial microbial community structure may influence the direction and extent of microbiota changes during diarrheal illness. According to your suggestion, we have revised the corresponding text in the Results section (Lines 221–224) and the Discussion section (Lines 439–446).

3. More interesting observations could be drawn if the PCoA analysis were done on taxa not only with the sample.
Thank you for this valuable suggestion. We further analyzed the PCoA results based on all genus-level taxa within the phyla Actinobacteria, Bacteroidetes, Firmicutes, and Proteobacteria, as well as the biomarkers identified in the more complex Cluster A. The results showed that the microbial differences between the non-diarrhea and diarrhea groups were driven primarily by genus-level compositional variation within Actinobacteria and Proteobacteria. In addition, *Clostridium* and *Neisseria* were identified as the key biomarkers of Cluster A in the non-diarrhea and diarrhea groups, respectively. The corresponding revisions have been incorporated into the Results section (Lines 264–268 and 280–282) and the Discussion section (Lines 446–453). The relevant visualizations have also been added as Supplementary Figure 2.

4. To target factors preventing disease, LEfSe should be done on the entire community.
Thank you for this valuable suggestion. We agree that LEfSe analysis of the entire microbial community can provide a more comprehensive view of taxa associated with the non-diarrheal state. Therefore, in the revised manuscript, we performed LEfSe analysis on all genus-level taxa in the non-diarrhea and diarrhea groups. This analysis was used to identify community-wide biomarkers that differed between the two groups. The corresponding results have been incorporated into the Results section, and the related visualization has been added as Figure 1g. Meanwhile, we also revised the related wording in the Results section (Lines 261–263).

Minor

1. Figure S4: Samples can be clustered, as it is difficult to interpret.

According to the reviewer's request, the heatmap in Figure S4 has been revised and clustered.

2. Biomarker analysis is packed with multiple concepts that could have been simplified.

Thank you for this helpful comment. To simplify the presentation, we revised the relevant wording throughout the manuscript. Specifically, LEfSe-derived results are now described as discriminative taxa or candidate biomarkers associated with specific groups, whereas RF-derived variables are described as predictive microbial features. We also removed or weakened wording implying protective effects, disease progression, rapid diagnosis, or treatment decisions where these interpretations were not directly supported by the analysis.

3. The accuracy and specificity of the random forest model should be compared with available random forest models.

Thank you for this valuable suggestion. In the revised Discussion, we have added a comparison with available studies to better contextualize the performance of our random forest model. For example, Brintz et al. developed predictive models for pediatric diarrheal etiology and reported a cross-validated AUC of 0.825 with a specificity of 0.85 for predicting viral etiology using a parsimonious clinical model. In addition, Boolchandani et al. used microbiome-based machine learning models to distinguish diarrheal from non-diarrheal samples and reported that the RF model achieved the best performance, with an accuracy of 84.9%.

In our study, the microbiome-based RF model for distinguishing bacterial from non-bacterial diarrhea achieved an accuracy of 82.62%, specificity of 85.23%, and AUC of 0.8485. These results indicate that the predictive performance of our model is generally consistent with previously reported models for diarrheal disease classification. We have added this comparison to the revised Discussion (Lines 502-507).

4. The RF model predicting pathogenic bacteria was low on accuracy.

We sincerely apologize that an incorrect set of results was inadvertently included in the manuscript. We have now replaced it with the correct version: an accuracy of 89.09%, recall of 90%, specificity of 88.10%, and AUC of 0.8788.

Reviewer #2

1. In lines 48-49 of the abstract, due to the lack of specific mechanism research in the study, conclusions regarding asymptomatic carriers should be inferred more cautiously.

Thank you for this important comment. We agree that, because this study mainly provides microbiome association data and does not include direct mechanistic validation, conclusions regarding asymptomatic pathogen carriage should be inferred more cautiously. In the original manuscript, some statements may have overemphasized the potential protective role of specific taxa. Therefore, we have revised the relevant statement in the Conclusions part (Lines 48-50) of the Abstract to avoid implying causality.

2. In Lines 166-169, the description of normalization for ARGs, MGEs, and VFs is unclear.

Thank you for this helpful comment. In the revised manuscript (Lines 187-190), we have clarified that the abundances of ARGs, MGEs, and VFs were normalized to the 16S rRNA gene abundance in each sample and expressed as copies per 16S rRNA gene. We also specified that the total

abundance of each functional gene category was calculated by summing the normalized abundances of all detected subtypes within that category.

3. In lines 266-270, the numbers and proportions related to proportions should be explained more clearly.

Thank you for this helpful comment. In the original manuscript, the numbers reported in Lines 266–270 referred to the total number of detected resistance-gene entries within each antibiotic resistance category across all samples in the corresponding group. For example, "tetracycline resistance genes, diarrhea: 777" indicated the total number of tetracycline resistance gene entries detected among all diarrhea-group samples.

However, we agree that this presentation was not sufficiently clear and could be misleading, as these counts are different from normalized ARG abundance and were not further used to support the interpretation of ARG distribution. Therefore, to improve clarity and avoid unnecessary confusion, we have removed this description from the revised manuscript.

4. In Section 3.5, since O2PLS can only be used for correlation analysis, the relevant statements regarding the inference of transmission pathways should be more cautious.

Thank you for this important comment. In the original manuscript, some statements in Section 3.5 may have overinterpreted the O2PLS results by implying specific routes of ARG and VF transmission. In the revised manuscript, we have modified both the section title and the related statements to focus on shared variation and association rather than transmission inference. Specifically, we now describe the results as showing that ARG and VF profiles were more strongly associated with MGE profiles than with broad bacterial community composition. The specific revisions have been made in the Results section (Lines 354–357) and the Discussion section (Lines 489–492).

5. In Section 3.6, the authors detected three non-bacterial pathogens. However, the use of the group name "bacterial pathogens with diarrhea symptoms (B-diarrheal subgroup)" in lines 337-338 may be confusing.

Thank you for this helpful comment. We agree that the original wording could be confusing, as the pathogen screening panel included non-bacterial pathogens, whereas the subsequent subgroup analysis was described using the term "bacterial pathogens." To avoid ambiguity, we have revised the relevant wording in Lines 359 and 362–367. In addition, because the main focus of this study was bacterial communities and bacterial pathogens rather than viral or parasitic pathogens, the subsequent analyses were restricted to samples carrying the relevant bacterial pathogens. To make this study purpose clearer and avoid potential misunderstanding, we further revised the wording in Lines 368–374.

6. In lines 407-413, since the supporting evidence is based on correlation analysis, the inferences regarding the protective capacity of Actinobacillus and Actinomyces for the host should be stated more cautiously.

Thank you for this valuable suggestion. We have revised the statements involving overinterpretation to make them more cautious and rigorous (Lines 454–461).

7. The description of the research plan for Limnohabitans in lines 416-419 is inappropriate.
Based on your suggestion, we have revised the original inappropriate wording in Lines 465–467.

Reviewer #3

1. In line 27, should it be 'Highlights' (possibly a typo)? Also, in the third bullet point, the sentence is missing a period at the end.
Thank you for pointing out this inappropriate wording. We have revised and corrected it accordingly.

2. In the Abstract (lines 33-38), the objective states "determine whether diarrhea is caused by bacterial infection," but the later description indicates a microbiome-based RF classifier. Please clarify in the Abstract whether the model predicts bacterial etiology defined by pathogen testing, or predicts diarrheal status/likelihood based on microbiome features.
Thank you for this helpful comment. We agree that the original wording in the Abstract could be misleading, as it might imply that the model was designed to directly determine whether diarrhea was caused by bacterial infection. In fact, the model was developed as a microbiome-based classifier to predict the potential etiology of diarrhea based on microbial community features. To clarify this point, we have revised the objective statement in the Abstract from "determine whether diarrhea is caused by bacterial infection" to "developed a microbiome-based model to predict the potential etiology of diarrhea." This revision more accurately reflects the purpose and scope of the RF classifier.

3. In the Introduction (lines 58-64), the statement "The primary treatment for severe acute diarrhea episodes is still antibiotic therapy" is overgeneralized. Please revise to a more cautious, guideline-consistent wording.
Thank you for this important comment. Following the reviewer's suggestion, we have revised this statement to provide a more cautious and guideline-consistent description (Lines 79-84). Specifically, we now emphasize that rehydration and maintenance of fluid–electrolyte balance remain the cornerstone of managing diarrheal disease in children, whereas antibiotics should be used selectively in cases with suspected or confirmed bacterial etiology. We also cited the relevant WHO guidance to support this revised statement.

4. In the Introduction (lines 77-84), "overt" and "covert" gene exchange are introduced as a key conceptual framework. Please add a brief definition or rephrase using standard terminology to avoid ambiguity.
Thank you for this helpful comment. To avoid confusion, we have revised this part using more explicit and standard descriptions of the possible directions of gene flow (Lines 99-105). Specifically, we now describe these processes as: (i) acquisition of ARGs by pathogens from the resident gut microbiota, and (ii) acquisition of ARGs or VFs by resident gut microbiota from external pathogens.

5. In Section 3.1 (lines 231-245), Cluster A/C are introduced with detailed phylum compositions, but their role in the narrative is not clearly stated at this point.
Thank you for this helpful comment. In the revised manuscript, we have added a clarification to indicate (Lines 264-268) that these clusters were used to characterize the inter-individual heterogeneity within the diarrhea group and to provide a basis for subsequent subgroup-level

comparisons.

6. In Section 3.2 (lines 253-264), "overall relative abundance" is used as the main descriptor, while Methods state that statistical evaluations are based on "normalized abundance values."
Thank you for this suggestion. We have updated and provided a more rigorous description of the abundance calculation in Section 2.4 of the Methods (Lines 187–190). We also revised the inappropriate wording in Section 3.2 accordingly.

7. In the Conclusion (lines 469-474), phrases such as "may play a crucial role in enhancing the probability of diarrhea" read as causal. Please revise to association-level language consistent with the evidence.
Thank you for this helpful comment. We agree that the original wording in the Conclusion may have implied a causal role of the identified gut microbiota in increasing the probability of diarrhea, which is not supported by the random forest analysis. The RF model identifies microbial features associated with diarrheal status or potential bacterial etiology, but it does not establish causality. Therefore, we have revised the relevant statement to more accurately reflect an association-based interpretation rather than a causal conclusion (Lines 521-526). Specifically, we now describe these taxa as gut microbial features associated with diarrheal status and potential bacterial etiology, and as candidate biomarkers that may help support etiological differentiation and appropriate antimicrobial decision-making, rather than as causal factors enhancing the probability of diarrhea.

8. In Figure captions, wording such as "samples positive for pathogenic" is grammatically incomplete. Please revise to "samples positive for pathogens/pathogenic microorganisms" and keep the caption language consistent with Section 3.6 terminology.
Thank you for the reviewer’s reminder. We have revised the relevant wording in Section 3.6 (Lines 362–371).

Re: mSystems00198-26R1 (**Comparative Gut Microbiome in Diarrheal and Non-Diarrheal Children: An Individually Matched Case-Control Study**)

Dear Dr. Xin Lu:

Thank you for the privilege of reviewing your work. Below you will find my comments, instructions from the mSystems editorial office, and the reviewer comments.

Please return the manuscript within 60 days; if you cannot complete the modification within this time period, please contact me. If you do not wish to modify the manuscript and prefer to submit it to another journal, notify me immediately so that the manuscript may be formally withdrawn from consideration by mSystems.

Revision Guidelines
To submit your modified manuscript, log into the submission site at https://msystems.msubmit.net/cgi-bin/main.plex. Go to Author Tasks and click the appropriate manuscript title to begin. The information you entered when you first submitted the paper will be displayed; update this as necessary. Note the following requirements:

• Upload point-by-point responses to the issues raised by the reviewers in a file named "Response to Reviewers," NOT in your cover letter.
• Upload a compare copy of the manuscript (without figures) as a "Marked-Up Manuscript" file.
• Upload a clean .DOC/.DOCX version of the revised manuscript and remove the previous version.
• Each figure must be uploaded as a separate, editable, high-resolution file (TIFF or EPS preferred), and any multipanel figures must be assembled into one file.
• Any supplemental material intended for posting by ASM should be uploaded with their legends separate from the main manuscript. You can combine all supplemental material into one file (preferred) or split it into a maximum of 10 files with all associated legends included.

For complete guidelines on revision requirements, see our Submission and Review Process webpage. Submission of a paper that does not conform to guidelines may delay acceptance of your manuscript.

Data availability: ASM policy requires that data be available to the public upon online posting of the article, so please verify all links to sequence records, if present, and make sure that each number retrieves the full record of the data. If a new accession number is not linked or a link is broken, provide mSystems production staff with the correct URL for the record. If the accession numbers for new data are not publicly accessible before the expected online posting of the article, publication may be delayed; please contact production staff (mSystems@asmusa.org) immediately with the expected release date.

Publication Fees: For information on publication fees and which article types are subject to charges, visit our website. If your manuscript is accepted for publication and any fees apply, you will be contacted separately about payment during the production process; please follow the instructions in that e-mail. Arrangements for payment must be made before your article is published.

ASM Membership: Corresponding authors may join or renew ASM membership to obtain discounts on publication fees. Need to upgrade your membership level? Please contact Customer Service at Service@asmusa.org.

The ASM Journals program strives for constant improvement in our submission and publication process. Please tell us how we can improve your experience by taking this quick Author Survey.

Thank you for submitting your paper to mSystems.

Sincerely,
Shrikant Mantri
Editor
mSystems

Reviewer #1 (Comments for the Author):

I appreciate the author's effort to address the reviewers' questions. Several critical aspects have been addressed, and efforts were made to further explore the community at a much deeper level. However, the manuscript still needs refinement to achieve clarity of statement, address the gaps in the analysis that could significantly enhance its contribution, and draw novel insights. A more focused exploration of the given comments will substantially improve the work to better align with the expectations of this

journal.
1. In response to the reviewer's question "How many days after diarrhea the stool sample was collected?" the author responded "1-2 days of symptom onset". This means that after diarrhea starts, within 1-2 days, the children were taken to the hospital. However, in the main article it is mentioned as "more than three loose or watery stools within a 24-hour period". It defines the confirmation of diarrhea but does not address the 1-2 day follow-up period mentioned in the response. This statement needs to be clarified.
2. The authors mentioned that they have reviewed the clinical record of the enrolled children and there was no prior administration of antibiotics. However, apart from antibiotic use, the study does not clearly indicate whether the children were receiving any prebiotic or probiotic treatment during this period.
3. The authors are advised to use abbreviations more cautiously, as some cases abbreviations were introduced without description. For example, Fig S1d, the description of CNF1 and CNF2 is missing.
4. The manuscript also requires precise use of terms, like "significance". In line 270-273 it was unclear how statistical significance was calculated, and the significance score should be highlighted.
5. In several instances, it was observed that data have been overlooked and adequate justifications were not provided. Despite differences in abundance of Actinobacteria and Firmicutes, why were they kept in a single cluster in Fig 1f? Why was only cluster A considered for biomarker analysis? Further PCoA analysis was performed between cluster A and cluster C; why not between the non-diarrheal and diarrheal community? An ordination biplot, plotting both samples and OTUs, can provide a better representation of how similar or distant the two communities are.
6. The clarity of figures is currently insufficient. For example, in Fig S4, the description of the color scale is missing. This figure further lacks granularity in terms of types of mobile genetic elements. A detailed characterization of each type - "IS element", "Transposase" could help generate deeper insights.

Reviewer #2 (Comments for the Author):

This is a meaningful metagenomic study on children's diarrhea, and the authors have provided detailed responses to each question.

Reviewer #3 (Comments for the Author):

The authors have thoroughly addressed all the concerns raised in the previous round of review. The manuscript has been significantly improved. I have no further comments.

I appreciate the author’s effort to address the reviewers’ questions. Several critical aspects have been addressed, and efforts were made to further explore the community at a much deeper level. However, the manuscript still needs refinement to achieve clarity of statement, address the gaps in the analysis that could significantly enhance its contribution, and draw novel insights. A more focused exploration of the given comments will substantially improve the work to better align with the expectations of this journal.

1. In response to the reviewer's question “How many days after diarrhea the stool sample was collected?” the author responded “1-2 days of symptom onset”. This means that after diarrhea starts, within 1-2 days, the children were taken to the hospital. However, in the main article it is mentioned as “more than three loose or watery stools within a 24-hour period”. It defines the confirmation of diarrhea but does not address the 1-2 day follow-up period mentioned in the response. This statement needs to be clarified.

2. The authors mentioned that they have reviewed the clinical record of the enrolled children and there was no prior administration of antibiotics. However, apart from antibiotic use, the study does not clearly indicate whether the children were receiving any prebiotic or probiotic treatment during this period.

3. The authors are advised to use abbreviations more cautiously, as some cases abbreviations were introduced without description. For example, Fig S1d, the description of CNF1 and CNF2 is missing.

4. The manuscript also requires precise use of terms, like “significance”. In line 270-273 it was unclear how statistical significance was calculated, and the significance score should be highlighted.

5. In several instances, it was observed that data have been overlooked and adequate justifications were not provided. Despite differences in abundance of Actinobacteria and Firmicutes, why were they kept in a single cluster in Fig 1f? Why only cluster A was considered for biomarker analysis? Further PCoA analysis was performed between cluster A and cluster C; why not between the non-diarrheal and diarrheal community? An ordination biplot, plotting both samples and OTUs, can provide a better representation of how similar or distant the two communities are.

6. The clarity of figures is currently insufficient. For example, in Fig S4, the description of the color scale is missing. This figure further lacks granularity in terms of types of mobile genetic elements. A detailed characterization of each type – “IS element”, “Transposase” could help generate deeper insights.

Reviewer #1

1. In response to the reviewer's question "How many days after diarrhea the stool sample was collected?" the author responded "1-2 days of symptom onset". This means that after diarrhea starts, within 1-2 days, the children were taken to the hospital. However, in the main article it is mentioned as "more than three loose or watery stools within a 24-hour period". It defines the confirmation of diarrhea but does not address the 1-2 day follow-up period mentioned in the response. This statement needs to be clarified.

Response:

Thank you for pointing out the unclear description in the manuscript. In this study, stool samples from all children with diarrhea were collected within 24 hours after the onset of the first diarrheal episode. We have further clarified the inclusion criteria and the timing of sample collection in Lines 130–135 of the revised manuscript.

2. The authors mentioned that they have reviewed the clinical record of the enrolled children and there was no prior administration of antibiotics. However, apart from antibiotic use, the study does not clearly indicate whether the children were receiving any prebiotic or probiotic treatment during this period.

Response:

Thank you for this question. Neither non-diarrheal children nor diarrheal children had any history of recent or long-term use of probiotics or prebiotics. The relevant information has been added to Lines 134–135 of the revised manuscript.

3. The authors are advised to use abbreviations more cautiously, as some cases abbreviations were introduced without description. For example, Fig S1d, the description of CNF1 and CNF2 is missing.

Response:

We sincerely apologize for this error. After rechecking Fig. S1d, we confirmed that “CNF1” and “CNF2” in the figure legend should have been labeled as “Cluster A” and “Cluster C,” respectively. This error has now been corrected.

4. The manuscript also requires precise use of terms, like "significance". In line 270-273 it was unclear how statistical significance was calculated, and the significance score should be highlighted.

Response:

Thank you for pointing out the imprecise use of the term “significant” in Lines 270–273. The original description of group separation was based on visual inspection of the PCoA plots rather than on a formal statistical test of between-group differences. Therefore, the wording implying significant separation was inappropriate. The relevant text has been revised in Lines 280. In addition, all uses of the term “significant” throughout the manuscript were carefully reviewed to ensure that each reported significance statement is supported by an appropriate statistical analysis.

5. In several instances, it was observed that data have been overlooked and adequate justifications

were not provided.

(1) Despite differences in abundance of Actinobacteria and Firmicutes, why were they kept in a single cluster in Fig 1f?

(2) Why was only cluster A considered for biomarker analysis?

(3) Further PCoA analysis was performed between cluster A and cluster C; why not between the non-diarrheal and diarrheal community?

(4) An ordination biplot, plotting both samples and OTUs, can provide a better representation of how similar or distant the two communities are.

Response:

(1) The UPGMA clustering shown in Fig. 1f was based on the overall similarity of microbial community composition and abundance across all detected taxa, rather than on the abundance of an individual phylum. Cluster A was therefore defined by the combined community profile and was characterized by the co-dominance of Actinobacteria and Firmicutes. Differences in the relative abundance of these two phyla did not require them to be assigned to separate clusters because the clustering reflected multivariate community-level distances.

(2) Cluster A was characterized by the co-dominance of Actinobacteria and Firmicutes, whereas Cluster C was overwhelmingly dominated by Proteobacteria. Although the non-diarrheal samples were mainly grouped in Cluster B, Cluster A still contained both diarrheal and non-diarrheal samples. Therefore, Cluster A provided a more informative and balanced background for identifying genus-level biomarkers associated with diarrheal status. In contrast, Cluster C consisted almost entirely of diarrheal samples and showed a very high proportion of Proteobacteria. Biomarker analysis within this cluster would therefore be strongly influenced by the dominance of Proteobacteria and would provide limited discriminatory information between diarrheal and non-diarrheal communities. For this reason, the biomarker analysis was focused on Cluster A.

(3) PCoA comparing the overall non-diarrheal and diarrheal communities had already been performed and is presented in Fig. 1e. The additional PCoA between Cluster A and Cluster C was conducted specifically to characterize the marked within-group heterogeneity observed among diarrheal samples.

(4) Thank you for this valuable suggestion. We agree that an ordination biplot can be useful for visualizing the relationships between samples and contributing taxonomic features. However, the present study was based on shotgun metagenomic sequencing rather than 16S rRNA amplicon sequencing, and no OTU clustering or OTU table was generated. Therefore, an OTU-based biplot could not be provided.

6. The clarity of figures is currently insufficient. For example, in Fig S4, the description of the color scale is missing. This figure further lacks granularity in terms of types of mobile genetic elements. A detailed characterization of each type - "IS element", "Transposase" could help generate deeper insights.

Response:

Thank you for this helpful comment. We have revised Fig. S4 and added a color scale, together with a clearer description of the figure legend and the normalization method used for visualization. This heatmap was intended to compare the relative abundance patterns of the four major MGE categories. The detailed MGE subtypes and their corresponding abundances are provided in Supplementary Table S6. In response to this suggestion, we have also added qualitative descriptions of transposases, plasmids, IS elements, integrase-related elements, and their representative subtypes in Lines 337–340 of the revised manuscript.

Re: mSystems00198-26R2 (**Comparative Gut Microbiome in Diarrheal and Non-Diarrheal Children: An Individually Matched Case-Control Study**)

Dear Dr. Xin Lu:

Your manuscript has been accepted, and I am forwarding it to the ASM production staff for publication. Your paper will first be checked to make sure all elements meet the technical requirements. ASM staff will contact you if anything needs to be revised before copyediting and production can begin. Otherwise, you will be notified when your proofs are ready to be viewed.

Data Availability: ASM policy requires that data be available to the public upon online posting of the article, so please verify all links to sequence records, if present, and make sure that each number retrieves the full record of the data. If a new accession number is not linked or a link is broken, provide production staff with the correct URL for the record. If the accession numbers for new data are not publicly accessible before the expected online posting of the article, publication may be delayed; please contact ASM production staff immediately with the expected release date.

Publication Fees: For information on publication fees and which article types have charges, please visit our website. We have partnered with Copyright Clearance Center (CCC) to collect author charges. If fees apply to your paper, you will receive a message from no-reply@copyright.com with further instructions. For questions related to paying charges through RightsLink, please contact CCC at ASM_Support@copyright.com or toll free at +1-877-622-5543. CCC makes every attempt to respond to all emails within 24 hours.

ASM Membership: Corresponding authors may join or renew ASM membership to obtain discounts on publication fees. Need to upgrade your membership level? Please contact Customer Service at Service@asmusa.org.

Embargo Policy: A press release may be issued as soon as the manuscript is posted on the mSystems Latest Articles webpage. The corresponding author will receive an email with the subject line "ASM Journals Author Services Notification" when the article is available online.

Cover Image Submissions: If you would like to submit a potential Cover Image, please email a file and a short legend to msystems@asmusa.org. Please note that we can only consider images that (i) the authors created or own and (ii) have not been previously published. By submitting, you agree that the image can be used under the same terms as the published article. Image File requirements: TIF/EPS, 7.5 inches wide by 8.25 inches tall (at least 2,250 pixels wide by 2,475 pixels tall), minimum 300 dpi resolution (600 dpi preferred), RGB, and no figure elements, e.g., arrows or panel labels. The legend should be a short description of the image, 1-2 sentences recommended. Please use the ASM Journals Cover Image Sizing Tool (https://journals.asm.org/pb-assets/html/asm-cover-sizing-tool.html) to ensure that your proposed cover image meets our size requirements.

Thank you for submitting your paper to mSystems.

Sincerely,
Shrikant Mantri
Editor
mSystems

Reviewer #1 (Comments for the Author):

The authors have thoroughly addressed all the concerns raised in the previous round of review. The manuscript has been significantly improved. I have no further comments.

Comments for the Authors,

The authors have thoroughly addressed all the concerns raised in the previous round of review. The manuscript has been significantly improved. I have no further comments.

Remarks for the Editors:

The authors have obtained ethical approval for the study involving human subjects between 1 to 5 years; however, there is no mention of patient consent. Were the parents informed about the study or the purpose of stool sample collection, and was written consent obtained before sample collection, needs to be mentioned in the manuscript.
