# Supplementary material for: Comparative gut microbiome in diarrheal and non-diarrheal children: an individually matched case-control study
Source: mSystems. 2026 Sep 30;11(10):e00198-26. doi: 10.1128/msystems.00198-26 (PMC13712888; doi:10.1128/msystems.00198-26)
Supplement: Supplementary file 2 [file msystems.00198-26-s0001.docx]

Fig. S1 Composition of Bacterial Communities in non-diarrhea and diarrhea group. (a) Comparison of Relative Abundances of *Escherichia*, *Salmonella*, *Actinobacillus*, and *Aggregatibacter* in non-diarrhea group and diarrhea group. (b) Relative comparison of microorganisms in Bacteroidia and Gammaproteobacteria between non-diarrhea and diarrhea group. (c) Bacteroidetes-to-Firmicutes ratio. (d) PCoA analysis of samples in Cluster A and Cluster C.

Fig. S2 PCoA of genus-level taxa within the phyla Actinobacteria (a), Bacteroidetes (b), Firmicutes (c), and Proteobacteria (d) in the non-diarrhea and diarrhea groups. (f) LEfSe analysis of all genus-level taxa in Cluster A from the non-diarrhea and diarrhea groups to identify the most important biomarkers.

Fig. S3 Comparison of relative abundance of virulence factors between non-diarrhea and diarrhea group.

Fig. S4 Heatmap of relative abundance of MGEs across all samples. The relative abundance of each MGE type was visualized after column-wise Z-score normalization. The color scale represents the standardized relative abundance within each sample, with warmer colors indicating relatively higher abundance and cooler colors indicating relatively lower abundance.

Fig. S5 Detection of common diarrheal pathogens by PCR. Red: negative; Blue: positive.
